# Seismic reinforcement of a linac installation

**DOI:** 10.1002/acm2.13214

**Published:** 2021-03-08

**Authors:** Martin Szegedi, Vikren Sarkar, Adam Paxton, Prema Rassiah, Bill J. Salter

**Affiliations:** ^1^ Department of Radiation Oncology University of Utah Salt Lake Utah USA

**Keywords:** seismic, linac installation, vault installation

## Abstract

The local building requirements to secure medical equipment in seismically active areas in the United States are based on recommendations of the American Society of Civil Engineers. In our institution we have recently acquired new linear accelerators, one of which had to be installed in an existing vault and one in a new vault. Since we are in a seismic active area, changes in the local code required us to start placing the new linacs seismically stable. Here, we describe the necessary steps taken to ensure a seismically sound installation of our linacs. For the linac installation to be seismically stable, the linac base frame has to be seismically fixed into the vault floor. The installation of a new linac into an existing vault requires verification of a structurally sound base frame. Knowledge of the previously applied fixation of such is needed and exploratory removal of grouted floor helped in the verification. Understanding the additional load requirements for the locality allows to account for the existing fixation and can potentially reduce the work needed to achieve seismic fixation requirements. For a prospective seismic installation the new linac base frame can be directly installed with the necessary strength. In addition the actual workflow is straight forward and vendor recommendations can be used. In both cases the vendor provided seismic calculations serve as baseline from which a facility should be work from. It is the facilities task to verify the correct installation of a linac in their specific location. An understanding of the seismic landscape can facilitate an appropriate installation at minimal additional cost.

## 1 INTRODUCTION

**Fig. 1:**
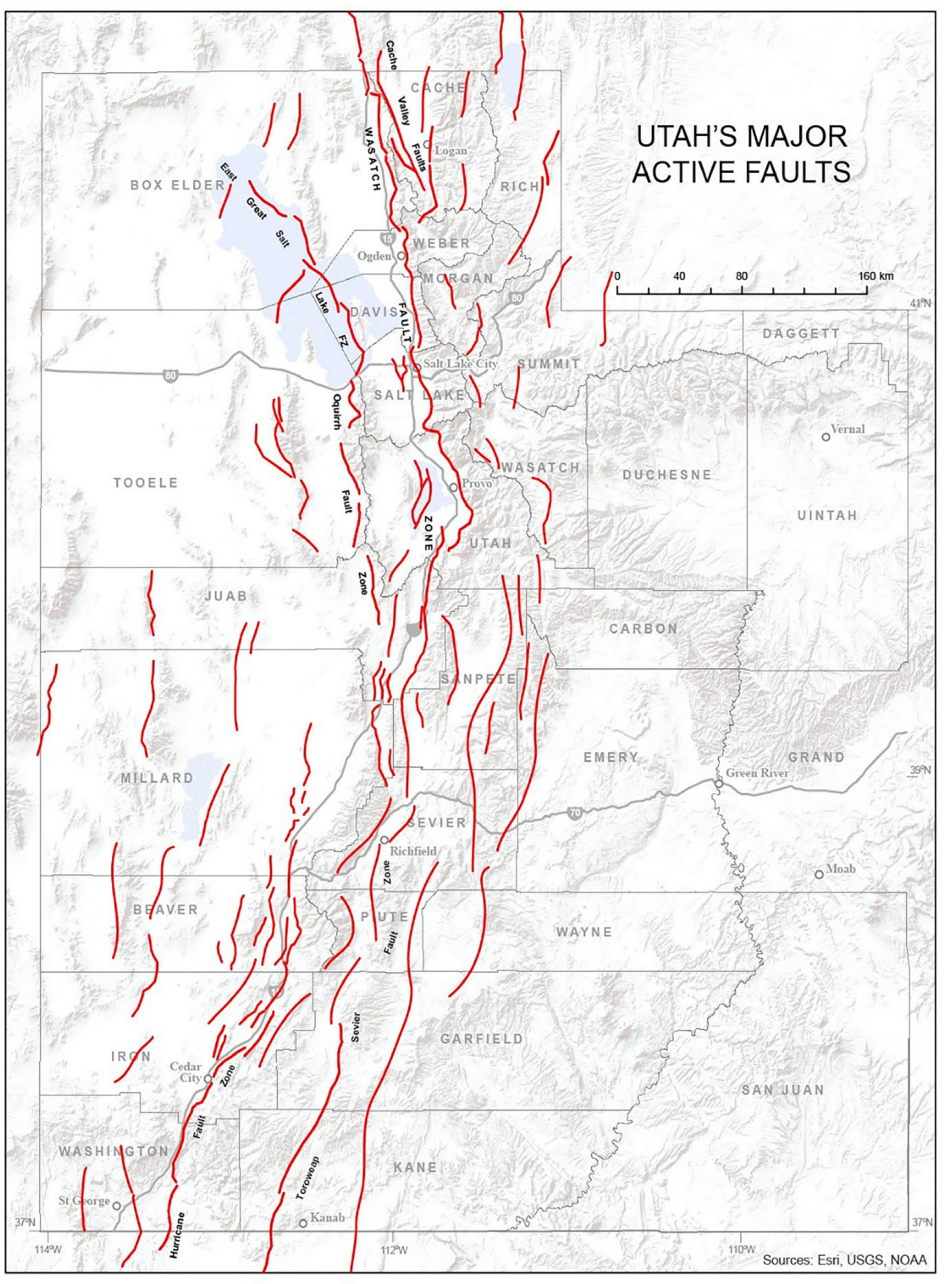
shows the active surface fault map of the Wasatch Front in Utah. Surface faults are shown in red.

Like many centers in the United States, the Huntsman Cancer Hospital of the University of Utah is located in a seismically active area, called the Intermountain seismic belt (ISB). Generally speaking, tectonic plate boundaries rarely run through continents, however, the ISB is an anomaly within the earth’s surface and lies within the interior of the North American plate.^1^ It is the transition zone between the relatively thin crust of the Basin and Range Province to the West and the thicker, more stable crust of the Rocky Mountains and Colorado Plateau to the east.^1^ Figure 1 shows a map of the surface faults of the ISB along the Wasatch Front of Utah.

Based on analysis of fault scarps (rock uprisings due to earthquakes) geologists have calculated a 50‐year earthquake probability of greater than 6.75 on the Richter scale in the Wasatch Front region of 43%, and a greater than 7.5 on the Richter scale probability of 15%.^2^ Such earth movement can cause surface displacements of up to 7.6 m (25 feet).

Therefore, along the Utah/Idaho Wasatch fault line, the potential for earthquake ground motions are similar to those expected in California. Of course, ground motion varies depending on specific location relative to the fault line, and expected forces are specified on maps published by the United States Geological Survey (USGS).

In the United States, the American Society of Civil Engineers (ASCE) updates structural requirements for building standards. The newest seismic recommendations are used and implemented into local building codes based on the USGS earthquake maps. Starting with the 2010 ASCE Standard 7–10 titled “Minimum Design Loads for Buildings and Other Structures”, there are specific requirements for non‐structural components, such as medical and lab equipment to be anchored to the building structure, i.e., typically fixation to the floor, with the anchorage strength based on the earthquake forces of the locality.^3^

In 2018, our institution began planning for two projects: (a) the replacement of a linac in an existing vault, using the existing grouted‐in base frame and (b) the construction of a new linac vault at a satellite facility. The ASCE’s latest seismic recommendations, which were signed into law by the Utah state legislature, were consulted during the planning phase. It was noted at this point that the current implementation of seismic fixations (i.e., the existing base frame) no longer met the updated state building code requirements. Since both projects were considered new implementations, the updated state requirements were required to be adhered to for both projects.

We believe that it may be unappreciated that many radiation therapy facilities are located in seismically active areas within the US and are therefore required to adhere to updated building codes. This work details the steps taken by our institution to address the seismic requirements for an existing vault (anchoring for an existing base‐frame) and also for implementing seismic anchoring in a new vault. Both of the cases presented here refer to anchoring of the Varian (Varian Medical Systems, Palo Alto, CA, USA) Universal Base‐frame (VUB); however, the basic principles of seismic fixation can be applied to any linac frame. We are hopeful that by presenting this work we can assist other departments in seismically active areas at being proactive in considering and possibly planning for seismic requirements in their next linac purchase, with the hope that delays and inefficiencies might be avoided, and safety might be improved.

## 2 DESCRIPTION

A licensed structural engineer was engaged in both cases to evaluate/design the anchoring of the equipment to the building structure based on anticipated local earthquake forces and the local code requirements for the anchors themselves.

### 2.1 Varian Universal Baseframe (VUB)

A linac installation (VUB, linac, and couch) will place more than 12,000 kg (26,500 lb) compressive force onto the vault floor. The VUB, which anchors this total weight to the floor, is made of steel, weighing 970 kg (2,138 lb) and, by itself, meets the stringent rigidity requirements of even the State of California.^4^ This frame is set in a recessed equipment pit, leveled, and bolted in place. The pit is then filled with grout around the VUB. Details of the pit dimensions are provided by the linac vendor. After being grouted in, the frame acts as the primary reference unit for the linac installation, essentially defining the location of machine isocenter.

**Fig. 2:**
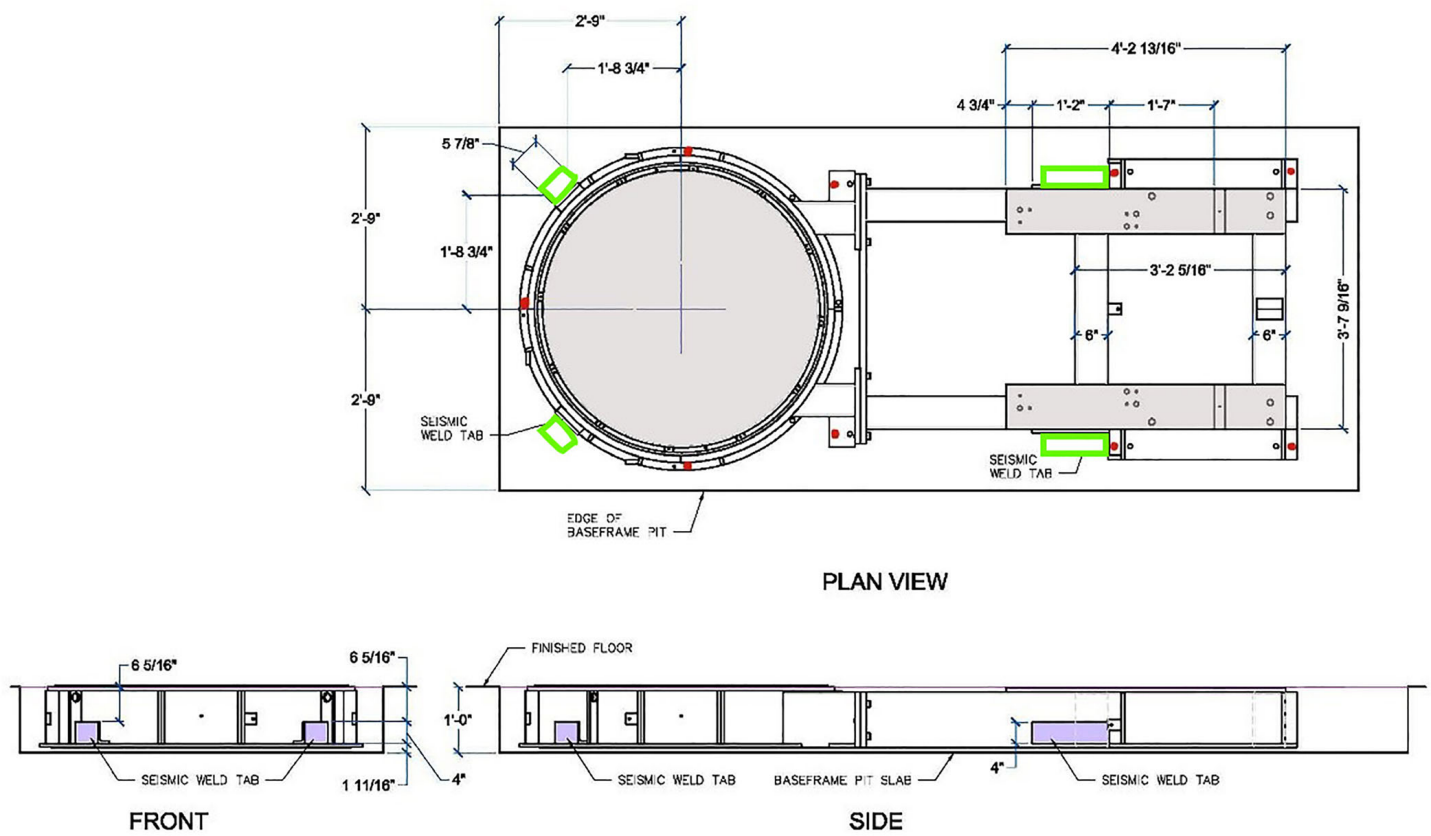
shows the VUB with main dimensions and standard bolt pattern (red dots) for fixing a VUB to a vault floor. The light purple colored welding tabs are visible in the side and front view. Couch Turntable Assembly and Stand Mounting pads are in light gray. L brackets as called out by the vendor are shown in light green next to the welding tabs in the Plan View.

Typically, a VUB is fixed in a 305 mm deep, 1676 mm wide pit, which runs at least 3760 mm long. The bolts used for securing the VUB to the pit follow a standard bolt pattern for non‐seismic linac VUB installation^5^ as shown by nine red dots in Fig. 2. The current standard bolts used are Bossard Expansion Anchors (BN 307, M10 threading) that have a recommended working load of 2500 N.^4^ Unfortunately, the design sheet does not specify tension or shear loads, likely because these bolts are intended to only hold the frame in place during grouting and to avoid VUB movement. While the grout is filled, it is pushed all around and under the frame, leaving only the inside of the VUB pull box and the underside of the Couch Turntable Assembly unfilled. Mechanical agitation is recommended to eliminate the chance of unfilled air pockets. Once grouted, the stand mounting pads on the top of the frame and the Turntable Assembly remain slightly above floor level, shown in Fig. 2 in light gray.

For a non‐seismic installation, assuming very conservatively that each bolt’s shear load is the same as the stated working load and accounting for nine anchors, the total load for this frame fixation is estimated to be about 2,250 kg, well below the 12,000 kg of weight for the whole linac installation. Thus for a prospective seismic VUB installation, the vendor‐provided seismic calculation^5^ does not include the standard 9 Bossard anchors and only calculates seismic load based on the 6 additional seismic anchors needed. The additional seismic bolt pattern using L bracketes, as called for by the manufacturer, is represented with light green rectangles in Fig. 2. Two of the seismic welding tabs for the VUB are located on the turntable side, at 51 degrees to each side of the longitudinal axis, having an area of 8 × 8 cm^2^. Larger side welding tabs are located near the stand mounting pad and have an area of 10 × 7.5 cm^2^ shown in light purple in Fig. 2.

### 2.2 Retrofitting of seismic anchoring to a VUB

In the case of the retrofit, the pit and anchoring of the VUB to the vault pit floor needed to be re‐evaluated. In our case, the specifications of the existing pit met the current state requirements for floor thickness and concrete reinforcements for the purpose of supporting the seismic installation of the VUB. The state required verification of existing building plans and installation documentation. The type of bolt, existence of seismic L brackets and/or welding pads on the VUB needed were not documented.

To minimize disruption and potential distortion/level to the existing VUB, one of the known fixation bolt locations closest to the strongest area of the baseframe (see Fig. 2) was chosen to visually confirm bolt type and weld‐pad availability. We measured levelness and concentricity of the VUB before the start of the project and after the testing/installation phase for consistency.

**Fig. 3:**
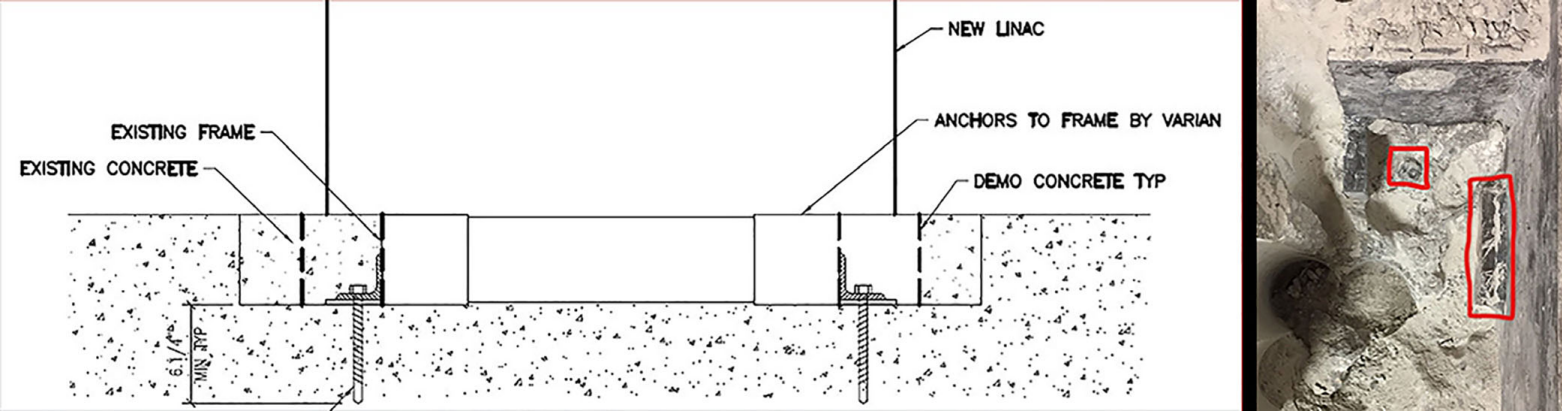
On the left is a cross section of the test hole created to evaluate the existing frame and anchors used. Next to it is a photo of the cleared frame, with a welding‐pad and bolt outlined in red.

Initially a 4” core‐drill and later a hand chisel were used to remove the flooring as needed to avoid damage to the existing baseframe. The first excavation established existing welding pad and dimensions. The fixation bolt type was the standard Bossard anchors. Figure 3 on the left shows the test hole as a cross section of the frame and an actual image. Local structural engineering calculations then considered the confirmed bolt loads and seismic forces to modify the Varian seismic calculations, requiring four additional smaller seismic L brackets using four high strength concrete anchors (Hilti KWIK HUS‐EX ¾) affixed to the VUB weld pads.

**Fig. 4:**
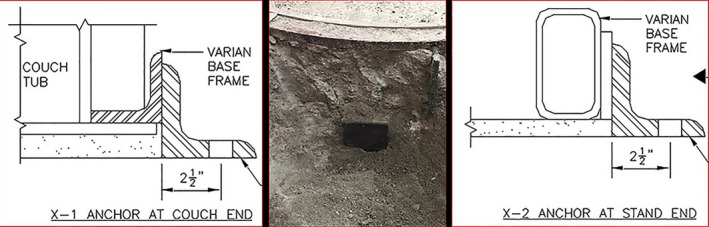
Technical drawing of the Varian base‐frame at the couch end with the 90° angle brace shown attached (left) and the actual location of the excavated Turntable Seismic Weld pad (center). On the right is a schematic detail drawing of the base‐frame Seismic Weld pad with 90° angle brace shown attached.

For the modification, we enlarged the existing test hole and added three similar sized holes in close proximity to where the baseframe weldpads were expected. Figure 4 shows an example excavation at the couch base and the technical drawings for excavation at the base‐frame and at the couch end.

**Fig. 5:**
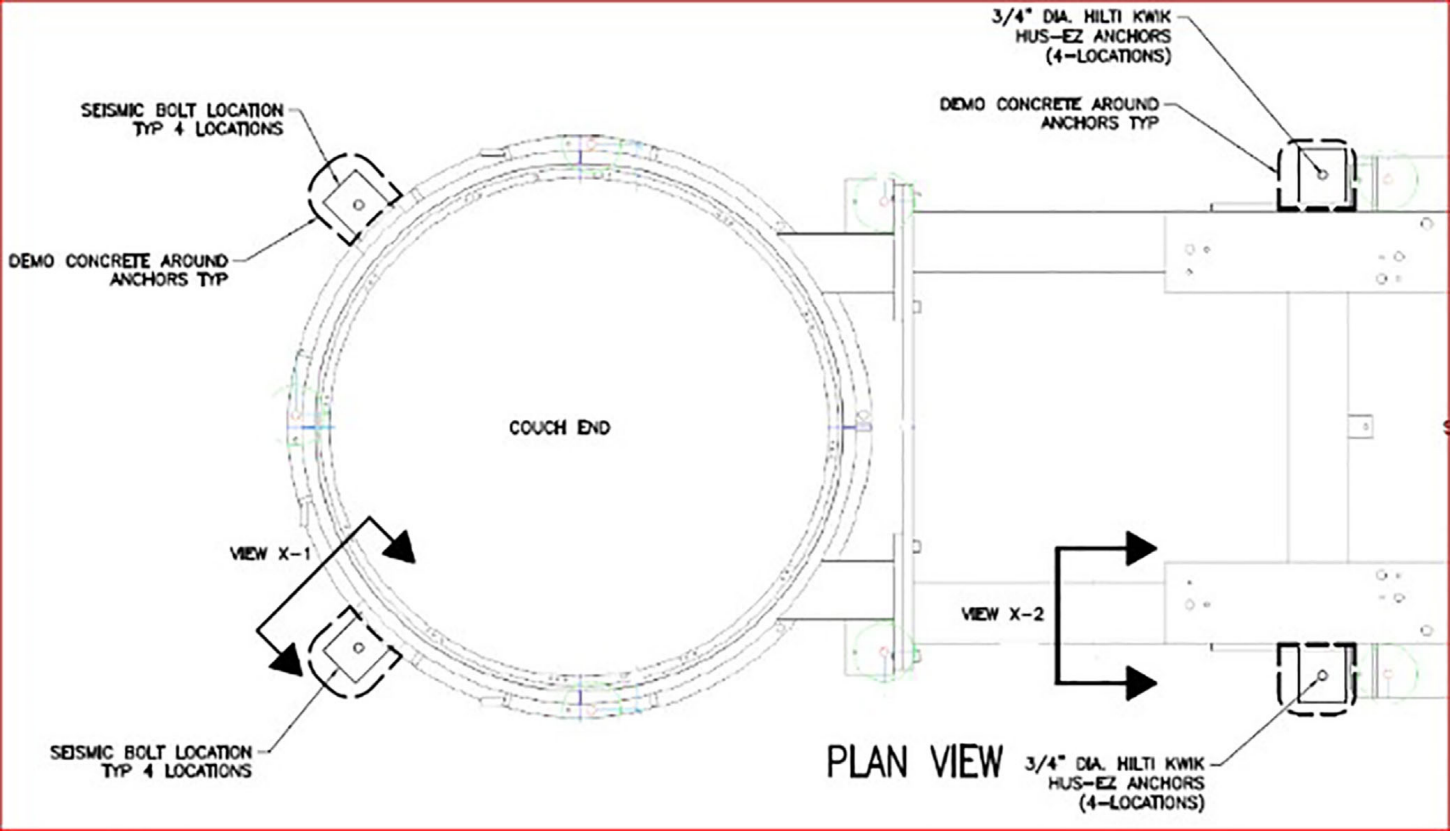
The inspected weld and bolted seismic fixation for the existing base‐frame.

**Fig. 6:**
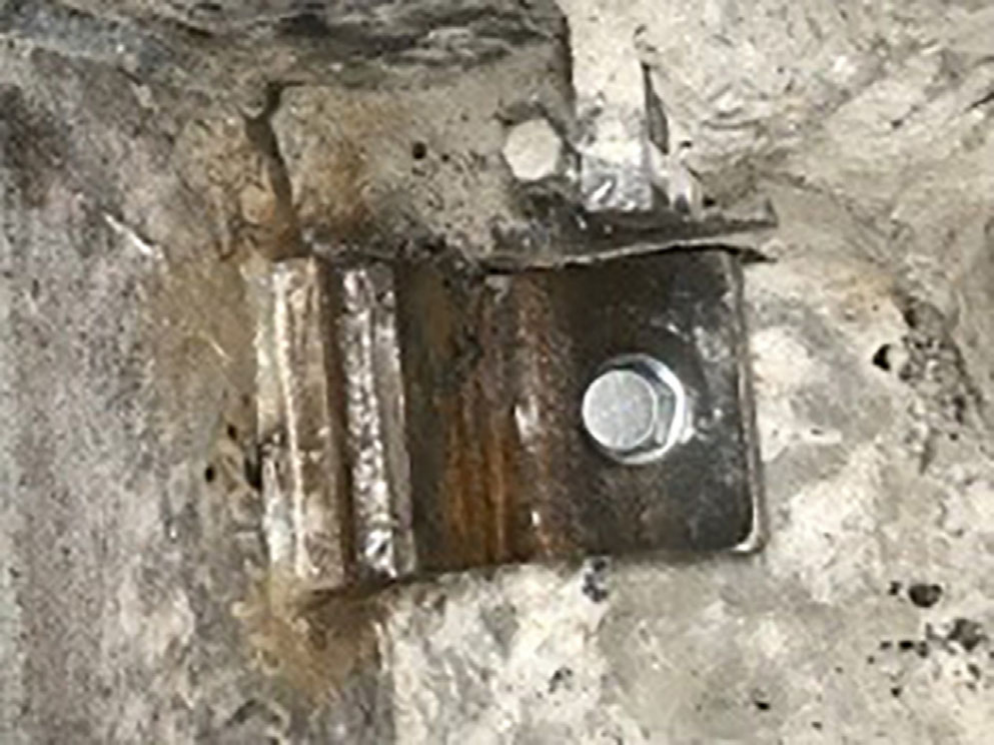
showing the final bolt pattern as installed to the existing VUB to achieve seismic code requirements. The required smaller angle irons and special anchors were sourced locally and installed under the observation of an official code inspector.

With enough room for welding and drilling work, the locally specified 90° angle braces were spot welded on. After spot welding, the Hilti anchors were placed as per installation requirements but not torqued to specification yet. In a final step, the L brackets were fully welded and bolts torqued to specification under the direct supervision of a state inspector who then approved the installation. Figure 5 shows one location after the inspection. Our solution, which fulfills local code is shown in Fig. 6. A final verification of the levelness of the turn table tub confirmed levelness and concentricity of the VUB. The floor holes were then grouted using greater than 3000 psi strength concrete.

### 2.3 Prospective fitting of seismic anchoring to a VUB

For the seismic anchoring of the VUB in the newly constructed vault, changes to the Varian sample seismic calculations^5^ were not needed, thus avoiding additional costs and making the actual workflow much more streamlined. In early 2020, we finished the construction of a new vault ensuring unimpeded access to the reinforced concrete VUB pit. After orienting and leveling the VUB it was fixed in place by the standard nine Bossard anchors. Subsequently we applied the vendor recommended process to seismically stabilize the VUB.

The Varian specified angle brackets^5^ were placed within 1 mm proximity to the weld‐pads of the frame and the floor was marked through the holes of the angle brackets. The floor was drilled in preparation of the Hilti M16 HAD‐T anchor placement. The angle brackets were then fixated by the torqued to specification anchors.

In order to avoid the frame being pulled by the welding (e.g., by heat deformation or structural deformation) all brackets were welded in a specific weld sequence, resulting in a three‐sided welded connection between the L bracket and the welding pad of the frame. Lastly, the welds and the bolt‐torque were inspected by a code inspector before the VUB was grouted in with above 3000 psi strength concrete.

## 3 DISCUSSION

Any earthquake of a substantial magnitude is likely to cause a radiation therapy department to stop treatments until all devices have been verified for function and accuracy. The purpose of additional seismic anchoring for a VUB is intended to prevent staff and patients in the room from being injured by dislodged and falling machinery during the actual quake.

It is up to each facility to understand and interpret the predictions of seismic maps for their specific location. Given that almost 50% of the continental US is considered ‘seismically active’, we believe that centers are well advised to carefully consider whether seismic anchoring is required/advised for your particular region. For prospective seismic anchoring, the material cost are generally less than $1,000 and labor amounted to roughly two man‐days, including inspection. The more involved retrofitting installation of seismic anchoring, which was deemed to be required for our site, introduced approximately $50,000 in additional cost. This total included engineering, special inspections, demolition, concrete cutting, welding, torqueing of bolts, patching the concrete, and repairing the floor. While it is clearly preferable from a cost and efficiency perspective to perform seismic anchoring prospectively, when necessary, retrofitting seismic bracing can be effectively accomplished.

## 4 CONCLUSION

Seismic activity for each institution’s region should be carefully considered when installing a new linear accelerator. If deemed necessary, seismic anchoring should ideally be performed upon replacement of a linac and base‐frame combination, as the workflow and cost are minimal for such installations. If required by local code, retrofitting strengthening of a linac base‐frame can be achieved for the specific environmental conditions of a particular site. The process should engage a licensed structural engineer and ensure a detailed documentation of all relevant information needed for proof of installation and for future reference.

## Author Contribution

Martin Szegedi was the lead physicist during vault refurbishment and vault building and as such responsible for supervision for the vault refurbishment and seismic frame verification and fixation. Dr. Salter joined during at critical times during the process for decision making in his capacity as the director of the department and. He and MS developed the idea of a publication given the novel challenges faced.

MS drafted an outline and did write the manuscript in consultation with VS, AP and PR. All authors provided critical feedback and helped shape the structure of the manuscript as well as the proof reading and formulation.

## CONFLICTS OF INTEREST

The authors report no conflict of interest for this publication.
